# Adhesion protein networks reveal functions proximal and distal to cell-matrix contacts

**DOI:** 10.1016/j.ceb.2016.02.013

**Published:** 2016-04

**Authors:** Adam Byron, Margaret C Frame

**Affiliations:** Edinburgh Cancer Research UK Centre, Institute of Genetics and Molecular Medicine, University of Edinburgh, Edinburgh, United Kingdom

## Abstract

Cell adhesion to the extracellular matrix is generally mediated by integrin receptors, which bind to intracellular adhesion proteins that form multi-molecular scaffolding and signalling complexes. The networks of proteins, and their interactions, are dynamic, mechanosensitive and extremely complex. Recent efforts to characterise adhesions using a variety of technologies, including imaging, proteomics and bioinformatics, have provided new insights into their composition, organisation and how they are regulated, and have also begun to reveal unexpected roles for so-called adhesion proteins in other cellular compartments (for example, the nucleus or centrosomes) in diseases such as cancer. We believe this is opening a new chapter on understanding the wider functions of adhesion proteins, both proximal and distal to cell-matrix contacts.

## Highlights

• Cell adhesion to the extracellular matrix (ECM) is vital for multicellular life.
• Complexes of structural and signalling proteins link the ECM to the cytoskeleton.
• Adhesion signalling networks are complex, diverse, dynamic and tightly regulated.
• Some adhesion proteins perform unexpected functions away from cell-matrix contacts.
• Nuclear roles for adhesion proteins have been revealed in diseases such as cancer.

## Graphical abstract

**Figure:**
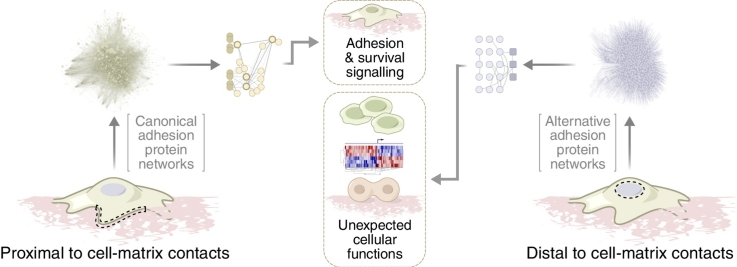

**Current Opinion in Cell Biology** 2016, **39**:93–100

This review comes from a themed issue on **Cell regulation**

Edited by **Manuela Baccarini** and **Ivan Dikic**

For a complete overview see the Issue and the Editorial

Available online 27th February 2016

**http://dx.doi.org/10.1016/j.ceb.2016.02.013**

0955-0674/© 2016 The Authors. Published by Elsevier Ltd. This is an open access article under the CC BY license (http://creativecommons.org/licenses/by/4.0/).

## Introduction

The extracellular matrix (ECM) forms an essential part of the cellular microenvironment; adhesion of cells to the ECM is critical for much of metazoan development, and its perturbation contributes to disease. The composition of ECM is highly diverse, containing proteins, glycoproteins and proteoglycans that interact to form a complex milieu [1]. It provides a structural support for cells to enable tissue formation and mechanosensing, and it binds soluble ligands and cell-surface receptors to trigger and coordinate cellular signalling [2]. Cells also use cell-surface adhesion receptors to sense the topology and stiffness of the pericellular ECM [3]. Mechanical information is transmitted via receptor-associated proteins to, and from, the actin cytoskeleton. Thus, adhesion receptors integrate and process biochemical and biophysical cues to control many aspects of cell behaviour, including differentiation, proliferation and migration.

The proteins that mediate adhesion signalling have been studied for decades. Recently, progress has been made in cataloguing the components of adhesions in various cell types, revealing that adhesion signalling is complex and diverse, both in terms of the number of components and the interrelations between them in signalling networks. Furthermore, the spatial restriction of this signalling is thought to drive emergent properties of multicellular systems in a way that is not yet fully understood [4]. Working out how cell adhesion systems function at a holistic network level is currently under intense scrutiny.

Here, we review recent progress in the elucidation of adhesion protein networks that mediate cell adhesion and provide the downstream effector signalling mechanisms. We also highlight new studies that have uncovered wider roles for adhesion protein signalling downstream of — and distal from — cell-ECM receptors. These studies suggest important new roles for adhesion proteins in diverse cellular locales.

## Adhesion signalling complexes: defining the players

The best-characterised family of cell-surface ECM receptors is the integrins, members of which interact with a range of ligands in the extracellular milieu [5]. Upon ligand binding, intracellular adhesion proteins are recruited to clustered integrin heterodimers at the plasma membrane, forming adhesion complexes [6, 7]. These consist of signalling and structural proteins that connect integrins to the actin cytoskeleton, the sum of which has been termed the ‘adhesome’ [8]. The latest literature-curated adhesome database contained 232 proteins derived from studies using multiple cell types and experimental conditions [9^••^].

**Figure 1:**
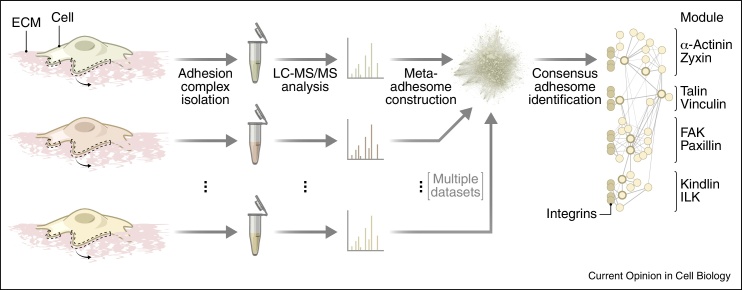
Definition of a consensus adhesome. Adhesion complexes induced by the integrin ligand fibronectin were stabilised and purified (curly arrows) and their proteomes were characterised by quantitative mass spectrometry (LC–MS/MS) in multiple studies using different cell types. Integration of these datasets generated a meta-adhesome, from which a core consensus adhesome was established [15••]. Network nodes (circles) represent interacting proteins; thick node borders indicate proteins that define the axes of emergent consensus adhesome modules (labelled, right).

Until recently, the comprehensive, global analysis of adhesomes was restricted by the challenges of purifying the labile, membrane- and cytoskeleton-linked adhesion complexes. The development of biochemical methodologies to isolate integrin-associated proteins, coupled with advances in proteomics and informatics, has largely overcome the earlier major challenges, thus enabling the characterisation of adhesion complexes by quantitative mass spectrometry [10, 11, 12, 13, 14]. Computational integration of multiple adhesion-site proteomes yielded an experimentally defined ‘meta-adhesome’, from which a core set of 60 frequently identified proteins — a ‘consensus’ adhesome — was identified [15^••^] (Figure 1).

It was clear from the first mass spectrometric analyses of isolated adhesion complexes that the number of proteins in these assemblies was greater than previously appreciated [16, 17, 18]. This showed that integrin-mediated adhesions are sites of considerable molecular complexity and diversity, and it is likely that they are sophisticated signalling hubs with physical and functional links to the cytoskeleton and to other organelles and cellular processes. Moreover, adhesion complexes induced by different extracellular ligands, or recruited to different integrin receptors, contain both common and condition-specific subsets of proteins [16, 19, 20]. Therefore, understanding the precise and context-dependent relationships between multiple adhesion proteins, and the mechanisms by which they control cell behaviour, have become important future priorities.

## Adhesion signalling close to integrins: mechanosensing the microenvironment

**Figure 2:**
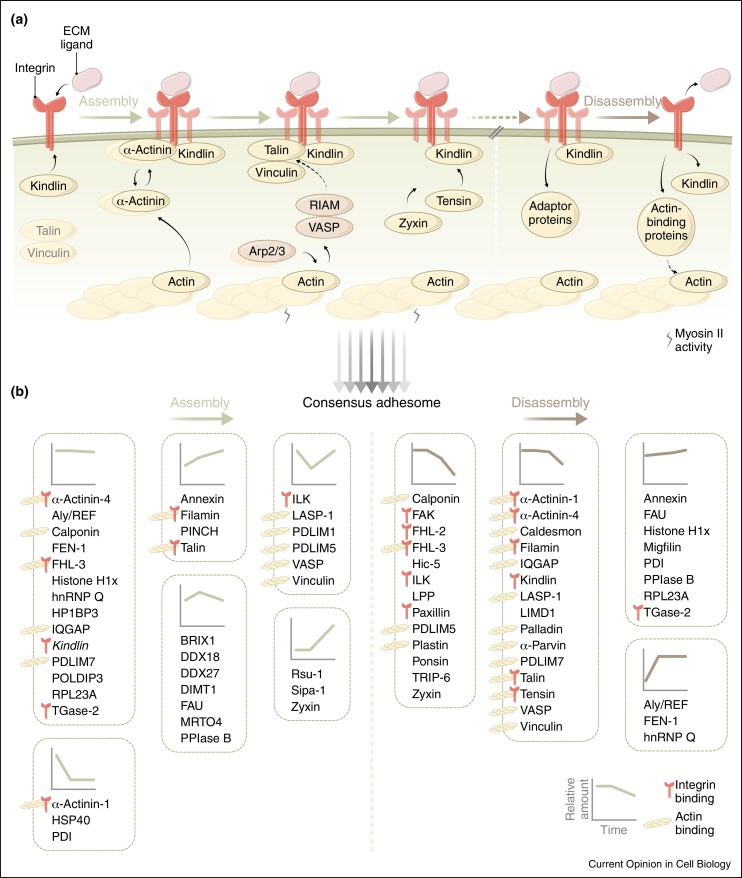
Temporal dynamics of adhesion complex composition. **(a)** Hierarchical recruitment of adhesion proteins to integrins (assembly, left), as determined by fluorescence microscopy studies. Some proteins may exist as pre-formed complexes in the cytoplasm (such as talin and vinculin, grey font). α-Actinin aggregates are transiently incorporated into developing adhesions and link integrins to the actin cytoskeleton. In membrane protrusions, RIAM binds Ena/VASP (brown nodes) and talin to link integrins to actin. Recruitment of talin to β1 integrin tails and maturation of adhesions requires myosin II activity, as indicated. Loss of adhesion proteins during disassembly (right), as suggested by proteomic experiments, also appears to occur hierarchically. **(b)** Assembly (left) and disassembly (right) dynamics of consensus adhesome proteins. Line profiles for each cluster show trends of protein abundance over time, as quantified by mass spectrometry [15^••^]. Integrin-binding and actin-binding proteins are indicated. Kindlin in the assembly dataset is kindlin-3 (italics), whereas kindlin-2 is the family member in the consensus adhesome.

The assembly and disassembly of adhesion complexes are tightly and dynamically regulated. However, the precise interactions of adhesion proteins are poorly defined in both space and time. A recent fluorescence correlation microscopic analysis of tagged adhesion proteins led to a model of hierarchical protein recruitment to integrins at early (nascent) adhesions [21^•^]. This proposed initial binding of kindlin-2 to α5β1 integrin, a role for α-actinin in nucleation of adhesions and subsequent association of talin and vinculin in response to myosin II activation (Figure 2). Talin forms a complex with vinculin before it associates with integrin [21^•^], as appears to be the case for several other adhesome components [22^•^]. Active myosin II generates mechanical forces that can change the conformation of proteins, including talin [23]. In filopodial and lamellipodial protrusions, talin links integrin to RIAM, which can promote actin polymerisation [24]. Focal adhesion kinase (FAK) may also accumulate at adhesion sites at the front of cells before paxillin [25], while some molecules, such as zyxin and tensin, are generally absent from nascent adhesions [26]. However, the temporal sequence of events may be cell and context specific. Proteomic quantification of assembly and disassembly of isolated adhesion complexes has revealed distinct temporal profiles of protein recruitment [15^••^]. These proteomic studies support the early recruitment of α-actinin and the later appearance of zyxin at adhesion sites (Figure 2). Moreover, adaptor proteins are apparently lost from adhesion complexes more rapidly than actin-binding proteins during disassembly, suggesting a relatively late disruption of the integrin-actin connection during adhesion turnover [15^••^].

Despite the remarkable consistency of very early adhesion assembly, regardless of ligand density, rigidity or intracellular tension [27, 28•], the stability and growth of nascent adhesions are regulated by physical links to the cytoskeleton and are influenced by actin-associated proteins (for example, formins, septins and synaptopodins [29, 30, 31]). Microtubules also influence adhesion complex composition and dynamics [32•, 33, 34, 35, 36], with their targeting to adhesion sites being regulated by integrin activation state [37^•^].

The interactions of vinculin with talin and actin probably form the major mechanosensory module that controls adhesion site composition, organisation and stability [38, 39•, 40, 41], with a role also for FAK [42, 43, 44]. Proteomics experiments have identified many proteins, including a number with LIM domains, that are preferentially incorporated into more mature adhesions under myosin II-generated tension [15••, 17, 18], so increasing the repertoire of likely proteins involved in mechanotransduction [45, 46]. Particular integrins (for example, α5β1 and αV) selectively recruit proteins that mediate differential responses to force, indicating that receptor-specific and multiple modes of rigidity sensing exist [20]. The key mechanisms by which adhesion complex components, and their molecular interactions, enable cells to sense their microenvironment remain to be fully defined in the future.

## Unexpected adhesion proteins: old functions for new proteins

Bioinformatic interrogation of datasets of adhesion proteomes has identified a substantial number of proteins that were not previously recognised as adhesion-linked proteins, and these have a broad range of predicted cellular functions [12, 15••, 37•]. Among the ‘non-canonical’ adhesion components, translation regulators are frequently detected in adhesion proteomes, supporting a long-held view that local protein translation occurs at adhesion sites [47]. Proteins involved in cytokinesis have also been discovered in adhesion complexes (e.g. RCC2 [16], CDK1 [48^•^]), strengthening the links between integrins and cell division [49]. There may also be transient ‘moonlighting’ roles for these proteins in integrin-mediated adhesion that have not yet been explored [50].

## Adhesion protein roles far from integrins: new functions for old proteins

Classical adhesion proteins appear to perform unanticipated functions at non-adhesion cellular sites. For example, the integrin-binding protein kindlin-1 has been found at centrosomes, where it ensures correct assembly of mitotic spindles [51]. FAK, in association with paxillin, is also required for correct spindle orientation [52^•^]. It therefore seems that a number of proteins may link cell adhesion status with spindle assembly and so tight control of cell division.

**Figure 3:**
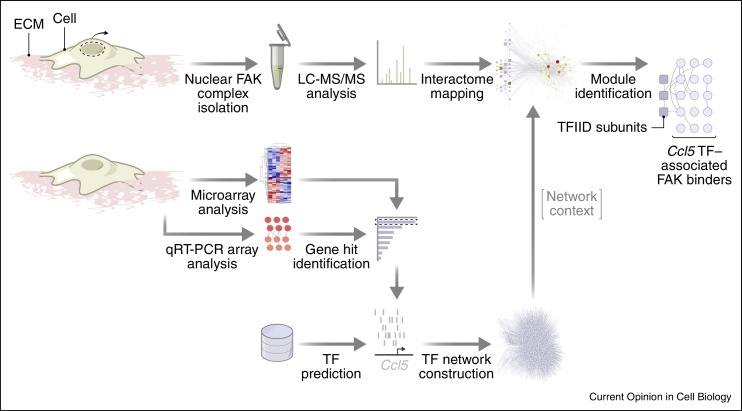
Integrative analysis of non-canonical FAK function. FAK complexes were isolated from purified SCC cell nuclei (curly arrow) and their interactomes were characterised by quantitative mass spectrometry (LC–MS/MS). The discovered protein interaction network was contextualised by mapping onto a network neighbourhood of transcription factors (TFs). Selected TFs were predicted to bind promoters of genes regulated by FAK (e.g. *Ccl5*), as identified by microarray and quantitative reverse transcription polymerase chain reaction (qRT-PCR) array analyses. This identified predicted TFs of FAK-regulated genes (e.g. transcription factor II D (TFIID) subunits) and their upstream regulators that interact with nuclear FAK [57^••^].

**Figure 4:**
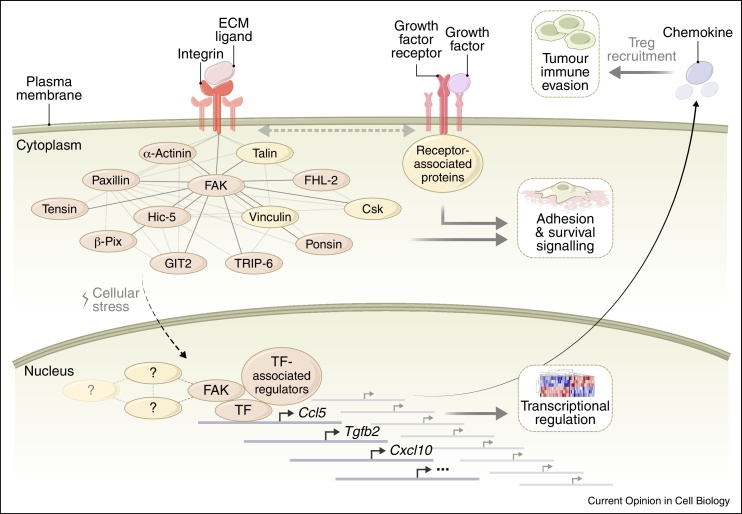
Role of FAK in the interplay between the microenvironment, adhesion receptor complexes and the nucleus. The network of FAK interaction partners from the consensus adhesome, and their predicted interactions (light grey lines; FAK interactions, dark grey lines), is shown associated with integrins at the plasma membrane. Synergistic integrin and growth factor receptor signalling regulates cell adhesion, migration and proliferation. Several FAK-interacting partners have been reported in the nucleus (brown nodes). Upon cellular stress, FAK localises to the nucleus, where it associates with chromatin and binds transcription factors and their regulators to control gene expression, including that of the chemokine Ccl5, leading to regulatory T cell (Treg) recruitment and immune evasion [57^••^].

FAK is also targeted to the nucleus (i) when normal cells receive cellular stress, where it can promote cell survival and regulate VCAM-1 expression [53, 54, 55], or alter heterochromatin organisation and promote muscle differentiation [56], and (ii) when it is overexpressed in squamous cell carcinoma (SCC) cells, thereby controlling the tumour microenvironment and immune evasion [57^••^]. Although FAK is predominantly visualised at focal adhesions by fluorescence microscopy, biochemical fractionation of SCC cells that express high levels of FAK revealed abundant nuclear FAK that we now know causes profoundly important effects on transcription [57^••^]. Indeed, FAK is associated with chromatin, and an integrative proteomic, bioinformatic and network approach discovered that FAK binds to components of the basal transcription machinery and upstream regulators of sequence-specific transcription factors that control chemokine production, exemplified by the FAK-regulated chemokine Ccl5 [57^••^] (Figure 3). While the detailed mechanisms by which FAK controls nuclear transcription of genes in a selective manner are still to be worked out, that study concluded that FAK scaffolds selective regulators of chemokine transcription, many in a kinase-dependent manner, in turn leading to regulatory T cell recruitment and immune evasion [57^••^] (Figure 4). The intermolecular interaction of the FAT and FERM domains of FAK is likely to be disrupted to reveal its nuclear localisation sequence [58, 59•], and nuclear FAK may remain monomeric, supporting its nuclear scaffolding role (and potentially hindering its observation in the nucleus by immunofluorescence). Importantly, there is no detectable nuclear FAK in normal skin keratinocytes [57^••^], implying that nuclear accumulation of FAK is linked to the cancer phenotype. This raises the possibility that nuclear functions of FAK, and potentially other adhesion proteins, could be specific to pathological states, such as cancer, providing potential therapeutic opportunities.

Other canonical adhesion proteins have been observed in the nucleus, e.g. zyxin, α-actinin and paxillin [60, 61, 62], and several have been shown to regulate hormone receptor signalling to influence gene transcription [63]. Indeed, the presence of a LIM domain appears to characterise a number of proteins that shuttle between integrin adhesion sites and the nucleus [64]. In addition, catenins, components of cell-cell adhesions, can enter the nucleus and control gene expression [65]. These findings raise a number of important questions about the generality, or specificity, of adhesion protein roles at sites far from adhesions. We do not know whether the role of nuclear FAK — or other nuclear adhesion proteins — is related to their better-understood adhesion roles (linking, for example, transcription to microenvironmental sensing) and, if so, how this is regulated or linked to disease phenotypes. It may be that some adhesion proteins have evolved scaffolding functions in other subcellular sites that are not related to their adhesion functions. The answers will undoubtedly come from further, ever more sophisticated, proteomic and network analyses, coupled to molecular intervention via genome editing and in physiologically relevant disease models. Nevertheless, these emerging data demonstrate a need to be cautious when addressing subcellular localisation solely by immunofluorescence microscopy, and they challenge our current view of the architecture and function of adhesion protein complexes. Moreover, they imply that long-range signalling by adhesion proteins, at sites distal to adhesions, will contribute to diverse cellular processes.

## Conclusions

For the coordination of a cellular process such as migration, networks of scaffolds and signals must combine to manifest a cohesive biological response. It is now clear that such molecular systems are extremely complex. Knowledge of the components that form the machinery of cell adhesion has become more complete over the past decade, and a consensus adhesome has now been defined. A universal adhesion ‘module’ appears to exist, comprising a set of ubiquitous, standard components, to which other modules of adhesion proteins associate and dissociate during adhesion site maturation and turnover. Such a modular system of protein complex assembly/disassembly and signalling could permit modulation of adhesion strength and rapid response to the ECM with fidelity. Of course, major questions remain about the precise molecular mechanisms that operate at the extracellular-intracellular interface, the ‘modular’ dynamics needed to respond rapidly to changing physical and chemical stimuli and transduce resulting signals, and the features that define context-dependent adhesion regulation and mis-regulation — such as in wound healing and disease. Immediate challenges have to include defining the roles of unexpected classes of molecules at adhesion sites and the roles of adhesion proteins at non-canonical, distal subcellular locations, and these represent active areas of investigation. Network analyses will provide systems-level answers.

## References and recommended reading

Papers of particular interest, published within the period of review, have been highlighted as:

• of special interest
•• of outstanding interest
